# Synchronous occurrence of benign mesothelioma and adenomatoid tumor of uterus: A case report and review of literature

**DOI:** 10.1097/MD.0000000000015746

**Published:** 2019-05-17

**Authors:** Ping-Rong Shen, Jin Cen, Xue-Qian Qian, Yuan-Ming Shen, Xiao-Dong Cheng, Xiao-Yun Wan

**Affiliations:** a Department of Gynecology, Maternal and Child Health Care Hospital of Ninghai County, Branch of Women's Hospital, School of Medicine, Zhejiang University; b Department of Gynecologic Oncology, Women's Hospital, School of Medicine, Zhejiang University, Hangzhou, China.

**Keywords:** adenomatoid tumor, benign mesothelioma, uterus

## Abstract

**Introduction:**

Synchronous occurrence of benign cystic mesothelioma and adenomatoid tumor of uterus (UAT) are very rare and few cases have been published in the English literature. They are easily misdiagnosed as malignant by clinicians, due to the lack of reports.

**Patient concerns:**

A case of benign mesothelial combined with uterus adenomatoid tumor (UAT) in a 48-year-old Chinese woman was reported. Our patient presented with abdominal pain and surgery showed a large subserous mass (12.0 × 11.4 × 9.8 cm) combined with a small intramural solid nodule (2.0 × 1.0 × 1.0 cm), and multiple minute neoplastic growth on the ovary.

**Diagnosis:**

Due to the patient's symptoms, pathological findings, she was diagnosed with synchronous occurrence of benign mesothelioma and UAT.

**Interventions:**

We treated her with a total hysterectomy and bilateral adnexectomy.

**Outcomes:**

The patient is now in stable condition, without any signs of recurrence during 1 year of follow-up.

**Lessons:**

Most mesotheliomas are malignant, synchronous occurrence of benign mesothelioma and UAT are extremely rare. And they are often misdiagnosed as malignancy by clinicians. Our case report can improve the awareness of the disease and avoid excessive treatment.

## 1 Introduction

Mesothelioma is a very rare tumor of mesodermal origin that covers the surface of the body's organs such as the peritoneum, pleura, and pericardium. Moreover, the malignancy accounts for a large majority, and its main characteristics present as increased cellularity, cytologic atypia, papillary formations, and even invasion. On the contrary, benign mesothelioma is much rarer, multicystic peritoneal mesothelioma (MPM), a type of benign ones, the incidence of which was reported only at 0.15/100000.^[1]^ In contrast to the malignancy, benign mesothelioma often has no history of asbestos exposure and may present as abdominal pain or mass. The lesions are limited, but have a high propensity for local recurrence and some reports suggested that adenomatoid tumors of the uterus may coexist.^[2]^ Clinicians often mistakenly believe that it is malignant, so there are often cases of overtreatment, including excessive surgical range, and unnecessary postoperative chemotherapy.^[3]^

To improve the clinicians’ awareness of the disease, here we reported a synchronous occurrence case of benign cystic mesothelioma and adenomatoid tumor of uterus (UAT). This case report was approved by the ethics committee of Maternal and Child Health Care Hospital of Ninghai County and Women's Hospital, School of Medicine, Zhejiang University. The informed consent form was signed by patient. The pathology was confirmed by Department of Pathology, Women's Hospital, School of Medicine, Zhejiang University.

## 2 Case presentation

A 48- year-old Chinese woman, gravida 4, para 2, was admitted to our hospital due to abdominal pain for 1 month. The clinical features of the patient at baseline were summarized in Table 1. The patient had a history of tubal ligation and denied asbestos exposure.

**Table 1:**
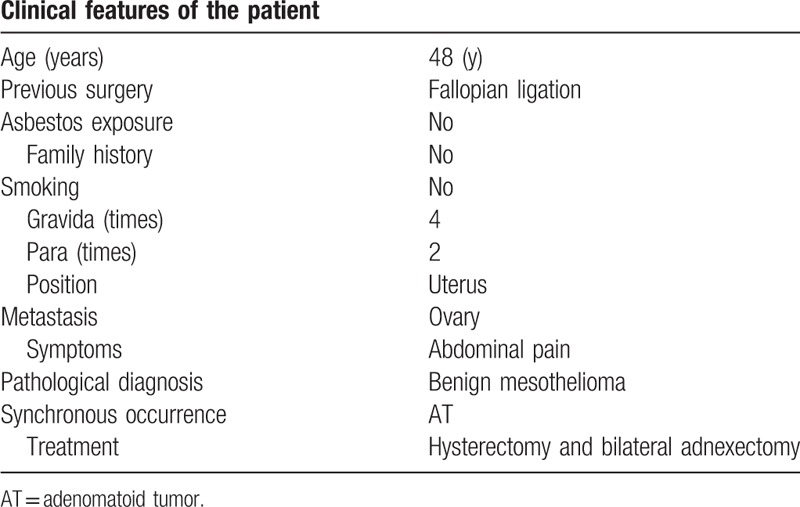
The clinical characteristics of the patient.

**Figure 1:**
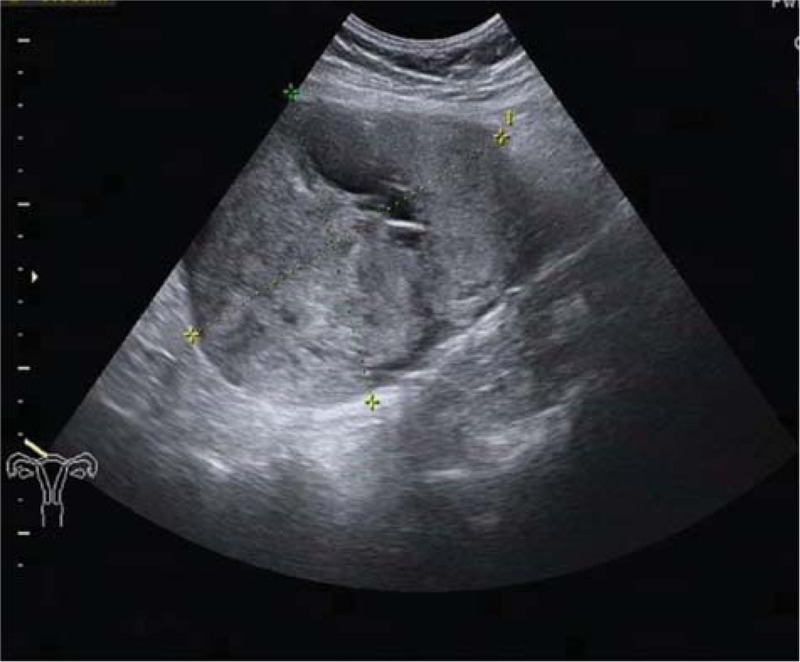
A pelvic mass examined by transvaginal ultrasound. Ultrasonography showed a 12 × 11.4 × 9.8 cm heterogeneous mass behind the uterus, with a cystic dark area of 3.4 × 2.3 cm.

**Figure 2:**
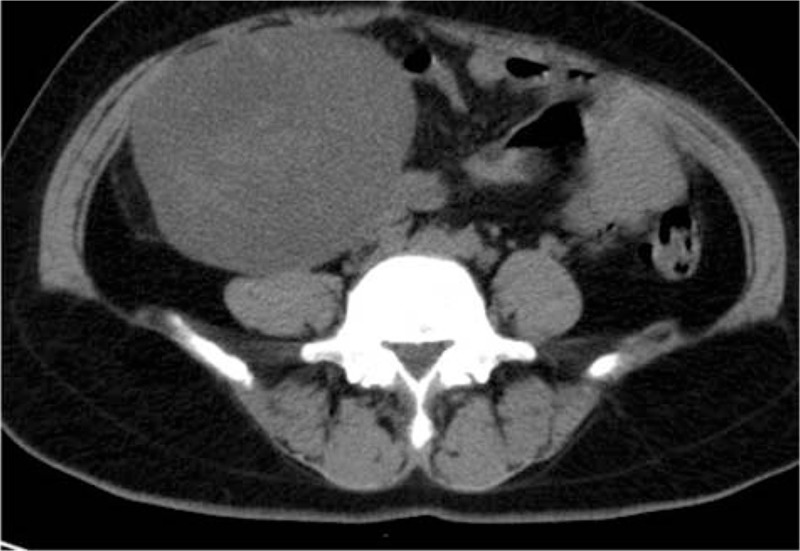
A pelvic mass examined by computed tomography (CT). CT examination revealed a well-defined mass in the posterior uterine region, which was considered fibroid steatosis.

Fifteen years ago, the patient was diagnosed with a uterine myoma, which was about 8 cm in diameter. Instead of undergoing surgery, she opted for regular check-ups. One month ago, she developed abdominal pain and ultrasonography showed a 12 × 11.4 × 9.8 cm heterogeneous mass behind the uterus, with a cystic dark area of 3.4 × 2.3 cm (Fig. 1). Ultrasound suspected as degeneration of uterine myoma. A further CT examination also considered it as a myoma fatty degeneration (Fig. 2). Therefore, the patient underwent exploratory laparotomy.

Intraoperative observations were described as below: There was a small amount of viscous fluid in the pelvic cavity. The uterus was enlarged to the size of 2 months gestation. There was a mass about 12 cm in diameter behind the uterus with a pedicle attached to the uterus. The mass was multilocular cystic with myxoid fluid. The surface of the left ovary was dotted with minute neoplasms. No obvious metastatic lesions were observed elsewhere.

**Figure 3:**
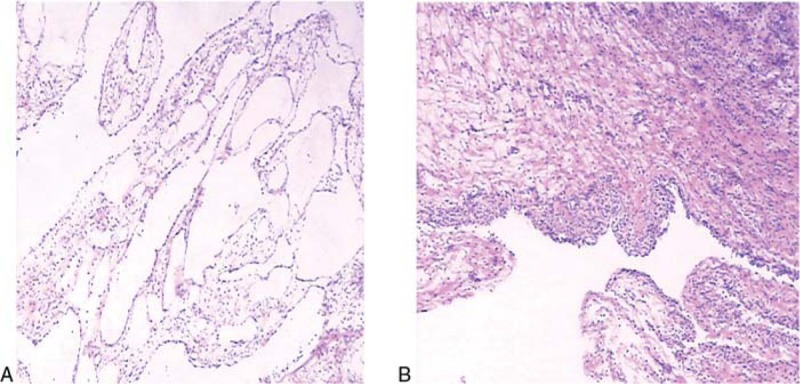
Pathological findings. (A) Benign mesothelioma (the cyst wall was lined by mesothelial cells without cytological atypia and invasion of underlying stroma). (B) Adenomatoid tumor (the tumor was consisted of epithelioid cells forming vacuoles and tubular spaces).

The mass was removed and a quick-freezing examination was performed. The results indicated that it was a mesenchymal tumor and adenomatoid tumor was possible. We treated her with a total hysterectomy and bilateral adnexectomy. The final pathology of paraffin confirmed it as localized well-differentiated benign mesothelioma (Fig. 3A). The results of immunohistochemistry showed that the 3 molecules Calretinin, D2–40, CK were positive, while p53, EMA were negative (Table 2). The tiny neoplasm on the surface of the ovary was also thought to be mesothelioma involvement. In addition, a small nodule with a diameter of about 2 cm was found in the myometrium, and the pathological result suggested adenomatoid tumor (Fig. 3B).

**Table 2:**
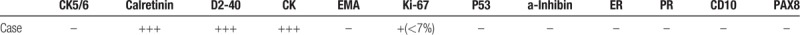
Immunohistochemical features of mesothelioma.

The patient is now in stable condition, without any signs of recurrence during 1 year of follow-up.

## 3 Discussion

Mesotheliomas are very rare neoplasms, representing a proliferative neoplasm made up of epithelial and mesenchymal cells, convering various organ surfaces within the body. Mesotheliomas can be generally divided into benign and malignant types, and benign ones are much rarer. As is well known, the malignant type is often associated with asbestos exposure. Yet, the exact etiology for the development of benign mesotheliomas remains unclear. Previous abdominal surgeries, trauma, alcohol use and smoking history, and family history were considered to be the possible reasons.^[4]^ Our patient had a history of tubal ligation, and no other significant risk factors were found (See Table 1). However, whether that was a direct factor, more cases remained to be accumulated.

How to distinguish between benign and malignant mesothelioma was still in progress.^[5]^ Radiology examination had limited value in the diagnosis of mesothelioma. Pathology would be the primary distinguishing evidence. Cytological atypia, dense cellularity, significant mitotic activity, invasion of underlying stroma and necrosis were always related to the malignant. Immunohistochemistry analysis is also becoming increasingly useful to guide the diagnosis. The molecules associated with malignancy include EMA, p53, glut-1, and IMP-3, while the benign molecules include Desmin, Calretinin, and D2–40.^[6–7]^ Our case presented as Calretinin, D2–40 positive and p53, EMA negative, which was consistent with the literature reports.

Through literature review, only 10 cases of benign mesothelioma have been reported so far. Hatano et al had already summarized 6 cases of benign mesothelioma combined with UAT in 2011.^[2]^ Here, we added 4 more benign mesothelioma cases, which were summarized in Table 3.^[8–11]^ Similar to those reported ones, our case also presented as abdominal pain, and no definitive history of asbestos exposure. Moreover, as in most cases, a hysterectomy was performed. However, it is worth mentioning that our case also has several unique aspects. Firstly, the onset age of our patients was 48 years old, higher than the common onset age (20–40 years old). Secondly, synchronous occurrence of UAT was detected postoperatively in pathology. AT is also a rare benign tumor, with the incidence of 0.04% to 1.74%,^[12]^ and its morphology is similar to that of adenoma, but its origin was considered as mesodermal.^[13]^ Adenomatoid tumors may coexist, suggesting that hysterectomy would be the appropriate choice.

**Table 3:**
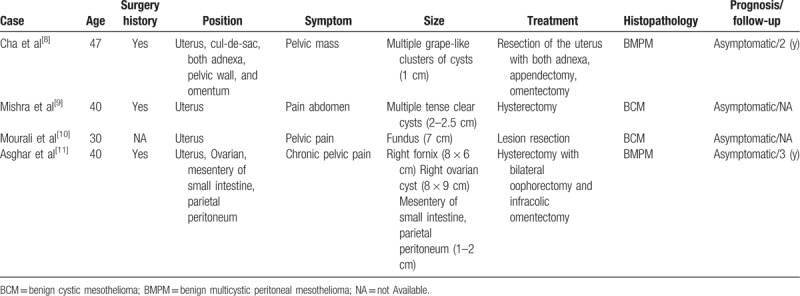
Summary of 4 reported cases of uterus benign mesothelioma in the English literature.

According to the literature review, postoperative chemotherapy was not generally recommended, although intraperitoneal chemotherapy has been reported in extremely rare cases.^[14]^ However, because of the risk of local recurrence, whether bilateral adnexectomy need to be performed during surgery requires further investigation, especially in young patients with ovarian metastases. Perhaps the patient's informed choice should be considered before a final decision was made. We treated our patient with a total hysterectomy and bilateral adnexectomy, after obtaining her informed choice. The patient did not receive any postoperative chemotherapy. And she is now in stable condition, without any signs of recurrence during nearly 1 year of follow-up.

## 4 Conclusion

Synchronous occurrence of benign mesothelioma and UAT are extremely rare and are easily misdiagnosed as malignancy by clinicians. Understanding these diseases can improve the skills of both clinicians and pathologist.

## Acknowledgments

We are grateful to the patient, who gave her informed consent for publication.

## Author contributions

**Data curation:** Jin Cen, Yuan-Ming Shen.

**Supervision:** Xue-Qian Qian, Xiao-dong Cheng.

**Writing – original draft:** Ping-Rong Shen.

**Writing – review & editing:** Xue-Qian Qian, Xiaoyun Wan.
